# Pregnancy Hypertension and a Commonly Inherited IGF1R Variant (rs2016347) Reduce Breast Cancer Risk by Enhancing Mammary Gland Involution

**DOI:** 10.1155/2019/6018432

**Published:** 2019-08-14

**Authors:** Mark J. Powell, Suzanne M. Dufault, Jill E. Henry, Anna C. Allison, Renata Cora, Christopher C. Benz

**Affiliations:** ^1^Buck Institute for Research on Aging, Novato, CA, USA; ^2^Zero Breast Cancer, San Rafael, CA, USA; ^3^Graduate Group in Biostatistics, University of California, Berkeley, School of Public Health, Berkeley, CA, USA; ^4^Susan G. Komen for the Cure® Tissue Bank at the Indiana University Simon Cancer Center, Indianapolis, IN, USA; ^5^Mission Analytics Group, San Francisco, CA, USA; ^6^Care Mount Medical, Mount Kisco, NY, USA

## Abstract

**Background:**

Terminal duct lobular units (TDLUs) are the anatomic sites of breast cancer initiation, and breast tissue involution resulting in lower TDLU counts has been associated with decreased breast cancer risk. The insulin-like growth factor (IGF) pathway plays a role in breast involution, and systemic changes in this developmental pathway occur with hypertensive disorders of pregnancy (HDP), which have also been associated with lower breast cancer risk, especially in women carrying a functional variant of IGF1R SNP rs2016347. We proposed that this breast cancer protective effect might be explained by increased breast tissue involution.

**Materials and Methods:**

We conducted a retrospective cohort study utilizing the Komen Tissue Bank, which collects breast tissue core biopsies from women without a history of breast cancer. Eighty white non-Hispanic women with a history of HDP were selected along with 120 nonexposed participants, and after genotyping for rs2016347, TDLU parameters were histologically measured blinded to participant characteristics from fixed biopsy sections.

**Results:**

Stratified models by HDP status demonstrated that among HDP+ participants, those carrying two T alleles of rs2016347 had a decrease in TDLU counts of 53.2% when compared to those with no T alleles (*p*=0.049). Trend analysis demonstrated a multiplicative decrease in counts of 31.6% per T allele (*p*=0.050). Although no statistically significant interaction was seen between HDP status and T alleles, interaction terms showed increasingly negative values reaching a *p* value of 0.124 for HDP × 2T alleles.

**Conclusions:**

The observed statistically significant decrease in TDLU counts signifies increased breast epithelial involution in women with prior HDP who inherited the TT genotype of IGF1R SNP rs2016347. The increasing degree of breast involution with greater rs2016347 T allele copy number is consistent with the known progressive reduction in IGF1R expression in breast and other normal tissues.

## 1. Introduction

Terminal duct lobular units (TDLUs) are the main structures within the breast that produce milk and are recognized as the anatomic site of development of most breast cancers [1]. Aging and the completion of childbearing are accompanied by mammary gland involution, and lower TDLU counts at a single point in time have been independently associated with lower breast cancer risk in many studies [2–4]. In addition, longitudinal data have shown that women whose breast tissue demonstrates slower involution over time also have increased breast cancer risk [5]. The insulin-like growth factor (IGF) pathway has been implicated in playing a role in the involution process, and decreased levels of IGF-1 and increased levels of one of its binding proteins, IGFBP3, have been associated with lower TDLU counts [6–8].

Hypertensive disorders of pregnancy (HDP) are also associated with systemic changes in the IGF pathway and affect later-life breast cancer risk. HDP impact 5–8% of pregnancies and are characterized by the development of high blood pressure usually after the 20^th^ week of pregnancy. HDP include gestational hypertension (hypertension alone) and preeclampsia (hypertension accompanied by proteinuria). These pregnancies are characterized by inadequate cytotrophoblastic invasion of the myometrium and impaired transformation of the spiral arteries resulting in placental ischemia [9, 10] and alterations in many hormones and growth factors including lower levels of IGF-1 and increased levels of IGFBP3 [11–15]. Many studies have reported lower breast cancer rates in women who experience HDP, and although these findings have not been uniform, most larger cohort studies have reported a decrease in later-life breast cancer rates ranging from 15–20% for both gestational hypertension and preeclampsia [16–18].

The breast cancer protective effect of HDP may have been underestimated in subgroups of women in prior studies that did not study inherited gene variants potentially affecting the IGF axis. Recent findings from the California Teachers Study demonstrated that among women with a history of preeclampsia, those carrying the TT genotype of a specific functional IGF1R SNP (rs2016347) had a decrease in risk for estrogen receptor-positive breast cancer of 74% when compared to the GG genotype [19]. Similarly, earlier work in the Marin Women's Study had found that in women with a history of HDP, carrying the T allele (allele frequency 0.52) was associated with lower later-life breast density as well as decreased breast cancer risk [20, 21]. This SNP is located in the 3' UTR of the IGF1R gene, and T alleles have been shown to result in a progressive decrease in IGF1R mRNA expression levels in breast and other normal human tissues [22].

Since the IGF pathway plays an essential role in early mammary gland growth and development as well as later-life breast tissue involution [23] and overstimulation of the IGF axis plays a promoting role in breast cancer development [24], we proposed that the profound breast cancer protective effect of HDP associated with inheritance of the IGF1R SNP rs2016347 TT genotype might be explained by and associated with increased breast tissue involution, manifested as lower TDLU counts. To test this hypothesis, we performed a retrospective cohort study evaluating TDLU counts from normal breast core biopsy samples from a cohort of genotyped parous women with no history of breast cancer.

## 2. Materials and Methods

### 2.1. Study Population

This retrospective cohort study utilized participants from the Komen Tissue Bank (KTB) at the Indiana University Simon Cancer Center. The KTB is an annotated biobank that collects breast tissue core biopsies, questionnaire data, and blood from women with no prior history of breast cancer and to date has received tissue donations from over 5,000 women. Donors provide written informed consent and are recruited under a protocol approved by the Indiana University Institutional Review Board. KTB participants were asked if they developed hypertension, gestational hypertension, or preeclampsia during a pregnancy and also if they had hypertension prior to pregnancy.

Eighty white non-Hispanic women were selected with a history of HDP if they answered yes to any of the questions about pregnancy hypertension and no to having had hypertension prior to pregnancy. One-hundred and twenty nonexposed participants were then selected from white non-Hispanic parous women who answered no to all questions, and these participants were frequency matched for age. The KTB provided digitized slides of the formalin-fixed and hematoxylin and eosin- (H&E-) stained biopsy sections on all participants along with reproductive history details and relevant covariates. Core biopsy tissue acquisition from an upper outer breast quadrant was standardized, and processing details are well described in the KTB standard operating procedures [25].

### 2.2. Genotyping

Upon entry into the study, blood was drawn from participants into an EDTA tube and after plasma separation and removal was stored at −80°C. Buffy coat DNA extraction occurred at the Indiana CTSI Specimen Storage Facility using an AutogenFlex Star instrument and Flexigene AGF3000 kit for DNA extraction. Genotyping for rs2016347 was performed at the Beckman Research Institute of City of Hope using MGB TaqMan Probe Assays from Life Technologies. The overall call rate was 97.0%, and the T allele frequency was 0.53 across the entire cohort.

### 2.3. Histologic Assessment

Histologic evaluation was performed by an experienced cytotechnologist (R. Cora) with specific training and expertise in assessing TDLU parameters and who was blinded to all participant characteristics and genotyping. H&E-stained digital images were reviewed using the Aperio ImageScope software from Leica (version 12.3.3); no samples contained either preneoplastic or malignant cells, and samples without any obvious epithelial component were considered ineligible for review. The mean tissue area scored was 35.26 mm^2^, and total counts of normal TDLUs were calculated per 100 mm^2^ and included any TDLU with at least 2 acini associated with a discernable lumen. Ninety-one of our participants had TDLU counts independently determined for another KTB study by Mayo Clinic pathologist M. Sherman, MD; these results were made available to us after our counts were completed and the paired readings showed high correlation, *r* = 0.89.

Nine of the 200 participants were not included in the final analysis due to lack of detectable epithelium on their biopsy slide (3) or inconclusive rs2016347 genotyping (6), resulting in an analytical dataset of 191.

### 2.4. Statistical Analysis

We ran negative binomial generalized linear models (GLM-NB) with a log-link to estimate adjusted count ratios (CRs) per unit of tissue area to assess whether TDLU counts varied with HDP status and whether this association was modified by IGF1R SNP rs2016347 T allele number. Models were adjusted for known confounders including age at first birth, age at time of biopsy, age at menarche, family history of a first-degree relative with breast cancer, body mass index (BMI), and parity. Different genotype parameterizations were used to test for trend and/or threshold effect on the TDLU counts. Multiplicative interaction was assessed via an interaction term in the GLM-NB between genotype and HDP at a significance threshold of 0.10.

Although there was a high percentage of zero TDLU counts (10%) despite presence of some epithelium, goodness-of-fit tests did not demonstrate improved fit for a zero-inflated or hurdle model when compared to the GLM-NB via the nested likelihood ratio test and the Vuong test for non-nested models, respectively. Additional nested goodness-of-fit testing compared the GLM-NB to the Poisson-GLM. This test returned evidence of improved model fit in the NB setting, suggesting overdispersion of TDLU counts. The model comparisons are summarized in supplementary [Supplementary-material supplementary-material-1].

All analyses were run using R version 3.5.0 “Joy in Playing” [26]. Estimation of the GLM-NB models was performed with the “glm.nb” function from the “MASS” package [27]. Zero-inflated models, hurdle models, and the Vuong goodness-of-fit test were estimated using “zeroinf,” “hurdle,” and “vuong” from the “pscl” package [28, 29]. All plots were made using the “ggplot2” package [30]. All code needed to recreate this analysis is available at https://github.com/sdufault15/tdlu-analysis.

Gail 5-year risk scores were calculated using the Breast Cancer Risk Assessment Tool located on the NIH website: https://bcrisktool.cancer.gov.

## 3. Results

### 3.1. Participant Characteristics

All participants were parous white non-Hispanic women by study design, and mean age at biopsy was 45.9 years. Characteristics of the major covariates are presented in Table 1.

HDP+ participants differed from HDP− participants only in having higher BMI (*p* < 0.001), with a mean BMI of 32.4 compared to 28.4 for HDP− participants. Obesity has been a frequently reported risk factor for HDP, and 55.2% of HDP+ participants in this study were obese (as defined by BMI >30) compared to 33.1% in the HDP− group [31, 32]. Mean values of all participants for parity, age at first birth, and age at menarche were 2.05, 27.0, and 12.5, respectively, and 26.7% reported a history of breast cancer in a first-degree relative.

### 3.2. Association of Breast Cancer Risk Characteristics and TDLU Counts

**Figure 1:**
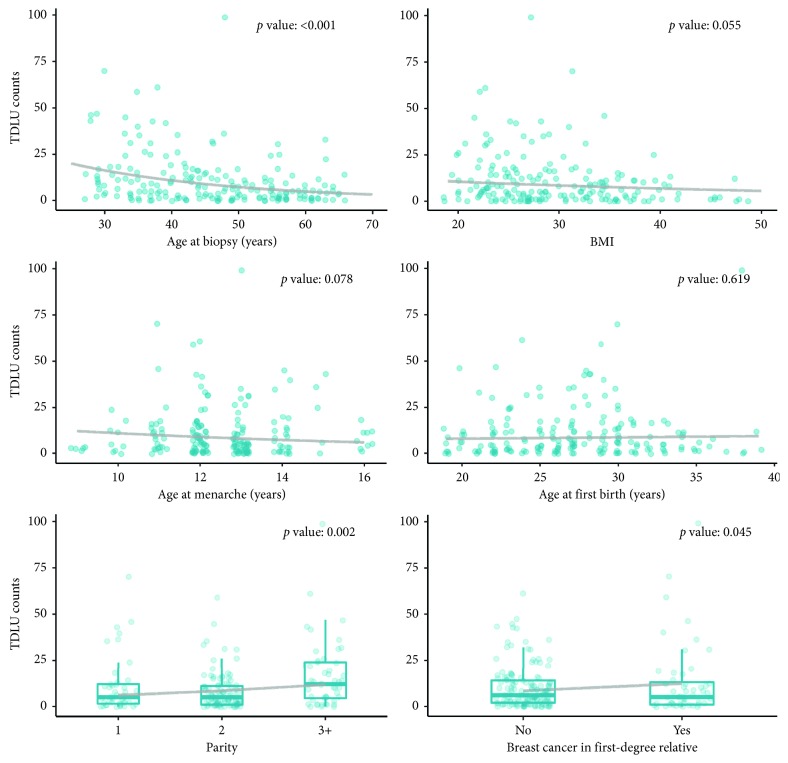
Adjusted bivariate TDLU relationships.

Relationships of major breast cancer risk factors with TDLU counts (adjusted for other covariates) are presented in Figure 1. Age at biopsy was inversely and significantly associated with TDLU counts, as would be expected. BMI was associated with lower TDLU counts, but this did not quite reach statistical significance (*p*=0.055). Both parity and family history of breast cancer were associated with increased TDLU counts, while there was little evidence of an association for age at menarche or age at first birth. In addition, Gail 5-year risk scores demonstrated no significant correlation with TDLU counts, *r* = −0.144.

### 3.3. Adjusted Negative Binomial Model for HDP and rs2016347 Genotype Interactions

When adjusted for multiple covariates associated with breast cancer risk, there were no statistically significant interactions between the effects of HDP status and the number of rs2016347 T alleles on TDLU count (Table 2), although Count Ratios (CRs) comparing the effects of 1 or 2 T alleles to 0 T alleles were, respectively, 0.734 (*p*=0.457) and 0.477 (*p*=0.124) times lower in the HDP+ stratum than in the HDP− stratum.

For women carrying no T alleles of rs2016347, the HDP+ exposure group has a TDLU count that is not significantly increased (CR = 1.23, *p*=0.546) when compared to the HDP− group. For HDP− women, the CRs comparing rs2016347 genotypes of 1 and 2 T alleles to the reference genotype of 0 T alleles show no evidence of association as both CRs hover around the null (CR = 0.973, CR = 1.104, respectively) with relatively large *p* values (*p*=0.918, *p*=0.747, respectively).

### 3.4. Box Plots of Adjusted TDLU Counts by rs2016347 Genotype Stratified by HDP Status

**Figure 2:**
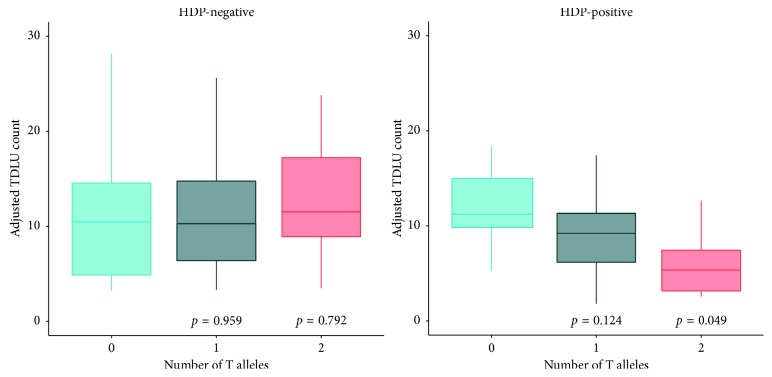
Box plots of adjusted TDLU counts by rs2016347 genotype stratified by HDP status.

The mean TDLU count across all participants was 11.01. Adjusted TDLU counts by rs2016347 genotypes stratified by HDP status are presented in Figure 2 (abbreviated model presented in Table 3 with full model in [Supplementary-material supplementary-material-1]). TDLU count distributions within the HDP− group were statistically similar across all genotypes; by contrast, within the HDP+ group, there was a stepwise decrease in TDLU counts with increasing rs2016347 T allele number reaching significance for 2 T alleles compared to 0 T alleles.

### 3.5. Adjusted Stratified Model and Trend Analysis by HDP Status and rs2016347 Genotype

Covariate adjusted models stratified by HDP status are shown in Table 3 and include factor models which look at the impact of 1 and 2 rs2016347 T alleles separately and a trend model which treats the T alleles linearly. In HDP− women, there is no effect of genotype on TDLU counts in either model. Among HDP+ participants, those carrying 2 T alleles showed a significant (*p*=0.049) decrease in TDLU counts of 53.2% when compared to those with 0 T alleles (GG genotype). Trend analysis in the HDP+ group also demonstrated a significant (*p*=0.050) linear trend with a multiplicative decrease in TDLU counts of 31.6% per T allele.

## 4. Discussion

In the current study, we were able to demonstrate a statistically significant decrease in TDLU counts, signifying increased breast epithelial involution, in women who have experienced HDP and inherited the TT genotype of IGF1R SNP rs2016347, while there was no evidence of an association for women who experienced either the TT genotype or HDP. This association with increased breast involution is very consistent with our prior findings in the California Teachers Study (CTS) where breast cancer incidence was similarly reduced in women with preeclampsia if they also inherited the TT genotype of rs2016347 but not when preeclampsia alone was considered [19]. In both studies, carrying one T allele produced an intermediate effect; furthermore, in the current study, the impact on breast involution increased according to T allele copy number consistent with the progressive reduction in IGF1R mRNA expression observed in breast and other normal tissues with increasing rs2016347 T allele number [22].

In our formal test for an HDP-genotype interaction, we observed that the CR comparing HDP+ to HDP− exposure groups carrying 2 T alleles of rs2016347 was 52.3% (*p*=0.124) lower than the CR comparing HDP+ to HDP− exposure groups carrying 0 T alleles of rs2016347, suggesting that the association of HDP with TDLU count is modified by rs2016347 genotype. Failing to achieve significant statistical interaction likely reflected our small number of HDP+ × 2T allele samples (21 of 191 total samples) and the extent of variance among sample TDLU counts. Nonetheless, the consistent pattern of association observed between this KTB analysis of normal breast TDLUs and our prior CTS analysis of breast cancer incidence rates strongly suggests that a history of HDP in concert with inheritance of a functionally blunted IGF1R rs2016347 variant manifests as both enhanced mammary gland involution and reduced later-life breast cancer risk, both outcomes impacted by decades of significantly reduced mammary gland IGF axis stimulation.

The IGF axis, stimulated primarily by soluble IGF-1 growth factor binding to and activating cell membrane-bound IGF1R growth factor receptor, plays a key role in breast development throughout life. Around the time of menarche, ovaries begin producing estrogen and progesterone resulting in expansion of the mammary ductal system with its stem and progenitor cell-enriched TDLUs. The IGF axis impacts this developmental process by the increase in circulating IGF-1 levels that accompany early menarche [33–35]. During pregnancy and with the onset of HDP, circulating IGF-1 levels are substantially reduced while IGFBP3 levels are increased (further reducing free IGF-1 levels), and as these reciprocal changes are sustained beyond parturition, they can attenuate IGF axis effects on the mammary gland and accelerate later-life breast involution [11–13, 20, 21].

Biologically, IGF-1 and IGFBP3 levels appear to drive only TDLU counts and do not otherwise impact other TDLU measures such as span or acini counts per TDLU [7, 8], consistent with our inability to detect either span or acini score associations with HDP and/or rs2016347 genotypes (results not provided). Across epidemiologic studies, HDP by itself only marginally reduces later-life breast cancer risk; but, as seen here and in two prior studies [19–21], inheritance of the functionally blunted rs2016347 TT genotype appears to combine with the biological impact of HDP to reduce mammary gland TDLUs, mammographic density, and later-life breast cancer incidence. By itself, higher expression of IGF1R in TDLUs can increase later-life risk of developing breast cancer by nearly 16-fold [36]. In contrast, among those women who ultimately develop breast cancer, inheriting the IGF1R expression blunting effect of the rs2016347 T allele confers a greater clinical response rate to neoadjuvant chemotherapy and a better overall survival outcome [37, 38].

Many well-established breast cancer risk factors can also independently impact TDLU counts, as shown in Figure 1, necessitating our multivariate model analyses (Tables 2 and 3) that adjusted for potentially confounding risk factors such as age at first birth, age at time of biopsy, age at menarche, breast cancer family history in a first-degree relative, BMI, and parity. We observed that age at biopsy was inversely associated with TDLU counts in a pattern similar to that reported by others, showing a declining slope with aging that does not change much after menopause [29]. TDLUs varied weakly and inversely with BMI; while this only trended toward significance (*p*=0.055), our findings are similar to what has previously been reported in other KTB cohorts [3, 39]. As with those other studies, we found that both parity and family history of breast cancer were associated with increased TDLU counts, while little consistent association was observed with regard to age at menarche or age at first birth. Likewise, Gail 5-year risk scores did not correlate with TDLU counts, consistent with findings from the Mayo Benign Breast Disease Cohort [40].

The use of specimens from the KTB provided a number of significant strengths to this study. First and foremost, it enabled assessment of entirely normal breast tissue donated by women without any history of breast cancer or other known breast disorders. Other “normal” breast studies commonly use resected tissue adjacent to breast tumors, biopsies taken for mammographically suspected breast lesions, or reduction mammoplasty samples. Furthermore, asking our participants if they had hypertension prior to pregnancy resulted in the exclusion of women with chronic hypertension, a cause of misclassification in many other HDP studies. In addition, the KTB has extensive data on reproductive history and other breast cancer risk factors, allowing us to account for many potentially confounding variables. Due to the relatively low number of women enrolled in the KTB at the time this study was initiated, we were limited in our ability to observe statistical significance for a moderate effect size, likely explaining the lack of statistical significance when formally testing for interaction between HDP history and rs2016347 genotype. We recognize that participants in the KTB are not completely representative of the general public, potentially limiting the generalizability of our findings. As such, it might be expected that women who volunteer for the KTB are more likely to have a positive family history of breast cancer, and this was noted for 26.7% of our study population, although women with BRCA1/2 positivity were excluded from our study cohort. Inclusion of only white non-Hispanic parous women was dictated by the low number of women of color enrolled by the KTB in its earlier stages.

## 5. Conclusions

Normal breast biopsy samples along with peripheral blood rs2016347 genotyping of 191 healthy parous female donors confirmed our mechanistic hypothesis that the pronounced breast cancer protective interaction between pregnancy hypertension (HDP) and inheritance of a functionally blunted IGF1R SNP (rs2016347) TT genotype likely results from enhanced breast glandular involution, as determined by fewer terminal duct lobular units (TDLUs).

## Figures and Tables

**Table 1:** Participant characteristics by HDP status^a^.

Characteristic	HDP+ *N* = 76	HDP− *N* = 115	*p* value^b^
	*N* (%)		
Age at biopsy (years)			0.82
≤39	26 (34.2)	36 (31.2)	
40–49	24 (31.6)	33 (28.7)	
50–59	18 (23.7)	29 (25.2)	
≥60	8 (10.5)	17 (14.8)	

Age at menarche (years)			0.23
≤11	19 (25.0)	19 (16.5)	
12	18 (23.7)	40 (34.8)	
13	25 (32.9)	31 (27.0)	
≥14	14 (18.4)	25 (21.7)	

Parity			0.38
1	17 (22.4)	26 (22.6)	
2	36 (47.4)	64 (55.7)	
≥3	23 (30.2)	25 (21.7)	

Age at first birth (years)			0.89
≤24	25 (32.9)	35 (30.4)	
25–29	26 (34.2)	43 (37.4)	
≥30	25 (32.9)	37 (32.2)	

BMI (kg/m^2^)			0.00
≤24.9	12 (15.8)	45 (39.1)	
25–29.9	22 (29.0)	32 (27.8)	
≥30	42 (55.2)	38 (33.1)	

Family history^c^			0.81
Yes	21 (27.6)	30 (26.1)	
No	55 (72.4)	85 (73.9)	

Rs2016347 genotype			0.83
GG	18 (23.7)	24 (20.9)	
GT	37 (48.7)	61 (53.0)	
TT	21 (27.6)	30 (26.1)	

Gail 5-year risk scores			0.44
Mean	1.50%	1.69%	

^a^All participants identify as non-Hispanic white. ^b^*p* value is the chi-squared *P* value for differences in distribution between HDP+ and HDP− participants. ^c^At least one first-degree relative with breast cancer.

**Table 2:** Results of adjusted negative binomial model with interaction terms.

	Coefficient	Standard error	Z Value	*p* value	CR (95% CI)
HDP	0.208	0.345	0.604	0.546	1.231 (0.627, 2.419)
T alleles = 1	−0.027	0.268	−0.102	0.918	0.973 (0.575, 1.645)
T alleles = 2	0.099	0.307	0.322	0.747	1.104 (0.605, 2.016)
HDP × T alleles = 1	−0.310	0.417	−0.743	0.457	0.734 (0.324, 1.661)
HDP × T alleles = 2	−0.740	0.482	−1.536	0.124	0.477 (0.185, 1.227)

HDP compares HDP-postive women to HDP-negative women. T alleles are treated as a factor variable. The reference for T alleles is no T alleles (T alleles = 0). These results are adjusted for family history, age at biopsy, parity, age at menarche, age at first birth, and BMI. Full model covariates can be found in the supplemental material ([Supplementary-material supplementary-material-1]).

**Table 3:** Summary table of the adjusted T allele CRs from the models stratified on HDP status.

	HDP-negative		HDP-positive	
	CR (95% CI)	*p* value	CR (95% CI)	*p* value
Factor model				
T alleles = 0	1 (ref.)		1 (ref.)	
T alleles = 1	1.014 (0.608, 1.689)	0.959	0.606 (0.320, 1.148)	0.124
T alleles = 2	1.083 (0.600, 1.955)	0.792	0.468 (0.219, 0.997)	0.049
Linear trend model				
∆ T alleles	1.042 (0.775, 1.400)	0.787	0.684 (0.468, 1.000)	0.050

These results are adjusted for family history, age at biopsy, parity, age at menarche, age at first birth, and BMI. Full model covariates can be found in the supplemental material (Tables [Supplementary-material supplementary-material-1] and [Supplementary-material supplementary-material-1]).

## Data Availability

The case and covariate data for this study were obtained from the Komen Tissue Bank (KTB). Generated data consisting of Gail scores, rs2016347 genotyping, and pathologic review of breast tissue samples with determination of TDLU parameters has been deposited in the KTB. All data for this analysis can be accessed on their virtual tissue bank at https://virtualtissuebank.iu.edu/.

## Supplementary Materials

Supplementary Materials Supplementary Table S1: goodness-of-fit model comparisons considered for this analysis. Supplementary Table S2: fully adjusted negative binomial model with interaction terms for HDP status and rs2016347 genotype, including covariates age at biopsy, age at first birth, family history of breast cancer in first degree relative, BMI, parity, and age at menarche. Supplementary Table S3: fully adjusted negative binomial models stratified for HDP status including covariates age at biopsy, age at first birth, family history of breast cancer in first degree relative, BMI, parity, and age at menarche. Supplementary Table S4: fully adjusted negative binomial model with alleles treated linearly for trend in both HDP-positive and HDP-negative participants including covariates age at biopsy, age at first birth, family history of breast cancer in first degree relative, BMI, parity, and age at menarche.
