# Supplementary material for: Pregnancy Hypertension and a Commonly Inherited IGF1R Variant (rs2016347) Reduce Breast Cancer Risk by Enhancing Mammary Gland Involution
Source: J Oncol. 2019 Aug 14;2019:6018432. doi: 10.1155/2019/6018432 (PMC6800903; doi:10.1155/2019/6018432)
Supplement: Supplementary Materials — Supplementary Table S1: goodness-of-fit model comparisons considered for this analysis. Supplementary Table S2: fully adjusted negative binomial model with interaction terms for HDP status and rs2016347 genotype, including covariates age at biopsy, age at first birth, family history of breast cancer in first degree relative, BMI, parity, and age at menarche. Supplementary Table S3: fully adjusted negative binomial models stratified for HDP status including covariates age at biopsy, age at first birth, family history of breast cancer in first degree relative, BMI, parity, and age at menarche. Supplementary Table S4: fully adjusted negative binomial model with alleles treated linearly for trend in both HDP-positive and HDP-negative participants including covariates age at biopsy, age at first birth, family history of breast cancer in first degree relative, BMI, parity, and age at menarche. [file 6018432.f1.pdf]

Supplementary Materials

TABLE S1: Goodness of fit test results

Alternative Hypothesis	p-value
GLM-NB > Poisson	0.000
ZIP > Poisson	0.000
ZINB > GLM-NB	0.660
GLM-NB > HNB	0.046

GLM-NB = generalized linear model with negative binomial distribution
Poisson = generalized linear model with Poisson distribution
ZIP = zero-inflated poisson model
ZINB = zero-inflated negative binomial model
HNB = negative binomial hurdle model

TABLE S2: Complete adjusted negative binomial model with interaction terms

	Coefficient	Standard Error	Z Value	p-value	CR (95% CI)
HDP	0.208	0.345	0.604	0.546	1.231 (0.627, 2.419)
T Alleles = 1	-0.027	0.268	-0.102	0.918	0.973 (0.575, 1.645)
T Alleles = 2	0.099	0.307	0.322	0.747	1.104 (0.605, 2.016)
AFB	0.008	0.017	0.498	0.619	1.008 (0.975, 1.043)
Age	-0.041	0.008	-5.232	0.000	0.96 (0.945, 0.975)
Family History	0.372	0.185	2.008	0.045	1.451 (1.009, 2.086)
BMI	-0.022	0.011	-1.918	0.055	0.979 (0.957, 1.000)
Parity	0.334	0.107	3.132	0.002	1.396 (1.133, 1.720)
Menarche	-0.102	0.058	-1.763	0.078	0.903 (0.806, 1.011)
HDP×T alleles = 1	-0.310	0.417	-0.743	0.457	0.734 (0.324, 1.661)
HDP×T alleles = 2	-0.740	0.482	-1.536	0.124	0.477 (0.185, 1.227)

T alleles are treated as factor variables. HDP compares HDP+ to HDP- women. The reference for T alleles is no T alleles (T alleles = 0). The reference for family history is no family history.

TABLE S3: Complete adjusted negative binomial models stratified by HDP status

	**For HDP+ women only**				
	Coefficient	Standard Error	Z Value	p-value	CR (95% CI)
T Alleles = 1	-0.500	0.326	-1.536	0.124	0.606 (0.320, 1.148)
T Alleles = 2	-0.760	0.386	-1.967	0.049	0.468 (0.219, 0.997)
AFB	-0.045	0.028	-1.628	0.104	0.956 (0.905, 1.009)
Age	-0.023	0.013	-1.793	0.073	0.978 (0.954, 1.002)
Family History	0.071	0.303	0.235	0.814	1.074 (0.593, 1.946)
BMI	-0.027	0.018	-1.522	0.128	0.973 (0.940, 1.008)
Parity	-0.048	0.179	-0.267	0.790	0.953 (0.671, 1.354)
Menarche	-0.224	0.095	-2.360	0.018	0.799 (0.663, 0.963)
	**For HDP- women only**				
	Coefficient	Standard Error	Z Value	p-value	CR (95% CI)
T Alleles = 1	0.013	0.260	0.052	0.959	1.014 (0.608, 1.689)
T Alleles = 2	0.080	0.302	0.264	0.792	1.083 (0.600, 1.955)
AFB	0.022	0.021	1.022	0.307	1.022 (0.980, 1.065)
Age	-0.043	0.010	-4.302	0.000	0.958 (0.940, 0.977)
Family History	0.441	0.229	1.924	0.054	1.554 (0.992, 2.435)
BMI	-0.020	0.014	-1.369	0.171	0.98 (0.953, 1.009)
Parity	0.398	0.131	3.028	0.002	1.489 (1.151, 1.927)
Menarche	-0.041	0.073	-0.556	0.578	0.96 (0.831, 1.109)

T alleles are treated as factor variables. The reference for T alleles is no T alleles. The reference for family history is no family history.

TABLE S4: Complete adjusted negative binomial models stratified by HDP status with alleles treated linearly for trend

	**For HDP+ women only**				
	Coefficient	Standard Error	Z Value	p-value	CR (95% CI)
T Alleles	-0.380	0.194	-1.960	0.050	0.684 (0.468, 1.000)
AFB	-0.046	0.028	-1.645	0.100	0.955 (0.904, 1.009)
Age	-0.022	0.013	-1.767	0.077	0.978 (0.954, 1.002)
Family History	0.103	0.300	0.342	0.733	1.108 (0.615, 1.996)
BMI	-0.028	0.018	-1.560	0.119	0.973 (0.939, 1.007)
Parity	-0.032	0.178	-0.182	0.856	0.968 (0.684, 1.371)
Menarche	-0.220	0.093	-2.375	0.018	0.802 (0.669, 0.962)
	**For HDP- women only**				
	Coefficient	Standard Error	Z Value	p-value	CR (95% CI)
T Alleles	0.041	0.151	0.270	0.787	1.042 (0.775, 1.400)
AFB	0.022	0.021	1.031	0.303	1.022 (0.981, 1.065)
Age	-0.043	0.010	-4.308	0.000	0.958 (0.940, 0.977)
Family History	0.436	0.228	1.911	0.056	1.547 (0.989, 2.419)
BMI	-0.020	0.014	-1.348	0.178	0.981 (0.953, 1.009)
Parity	0.402	0.131	3.071	0.002	1.495 (1.157, 1.933)
Menarche	-0.039	0.072	-0.550	0.582	0.961 (0.836, 1.106)

T alleles are treated linearly. The reference for T alleles is no T alleles. The reference for family history is no family history.
